# Supplementary material for: Simple and Highly Efficient Detection of PSD95 Using a Nanobody and Its Recombinant Heavy-Chain Antibody Derivatives
Source: Int J Mol Sci. 2023 Apr 14;24(8):7294. doi: 10.3390/ijms24087294 (PMC10138605; doi:10.3390/ijms24087294)
Supplement: Supplementary file 1 [file ijms-24-07294-s001.zip › ijms-2318109-supplementary.pdf]

Supplementary information

Simple and Highly Efficient Detection of PSD95 Using a Nanobody and Its Recombinant Heavy-chain Antibody Derivatives

Markus Kilisch[1], Maja Gere-Becker[1], Liane Wüstefeld[2], Christel Bonnas[2], Alexander Crauel[1], Maja Mechmershausen[1], Henrik Martens[2], Hansjörg Götzke[1], Felipe Opazo[1,3,4*], Steffen Frey[1*]

[1]NanoTag Biotechnologies GmbH, Rudolf-Wissell-Straße 28a, 37079 Göttingen.

[2]Synaptic Systems GmbH, Rudolf-Wissell-Straße 28a, 37079 Göttingen.

[3]Institute of Neuro- and Sensory Physiology, University Medical Center Göttingen, 37073 Göttingen, Germany.

[4]Center for Biostructural Imaging of Neurodegeneration (BIN), University of Göttingen Medical Center, 37075 Göttingen, Germany.

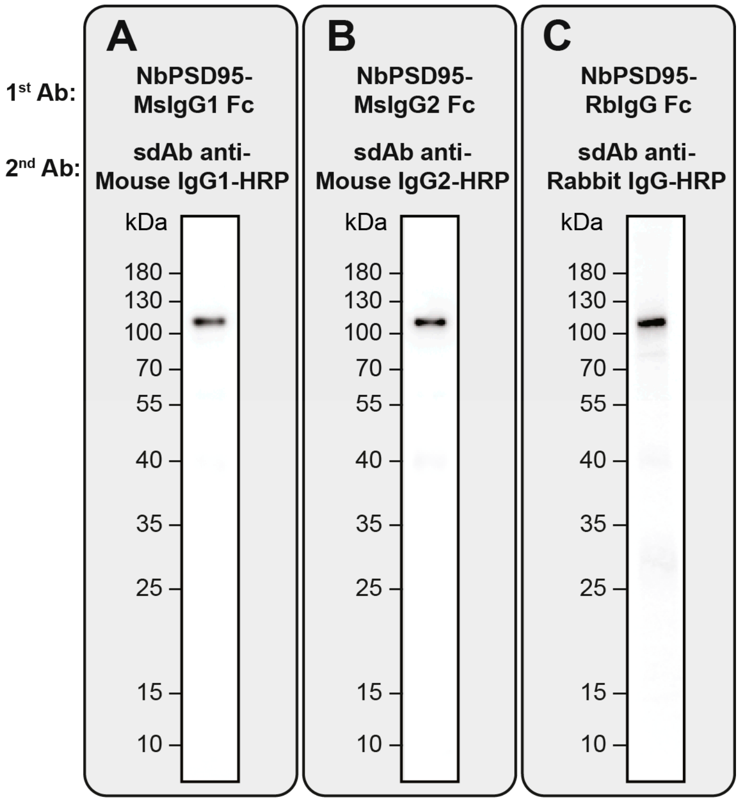

Figure S1: anti-PSD95 r-hcAbs harboring different Fc domains specifically recognize PSD95 in Western blot applications.

Total mouse brain lysates were analyzed by Western blot using r-hcAbs consisting of NbPSD95 fused to a Fc domain from either mouse IgG1 (MsIgG1 Fc; **A**), mouse IgG2 (MsIgG2 Fc; **B**) or rabbit IgG (RbIgG Fc; **C**). Detection was performed using HRP-coupled secondary nanobodies recognizing the respective Fc domains. All three r-hcAbs detected PSD95 with similar specificity.

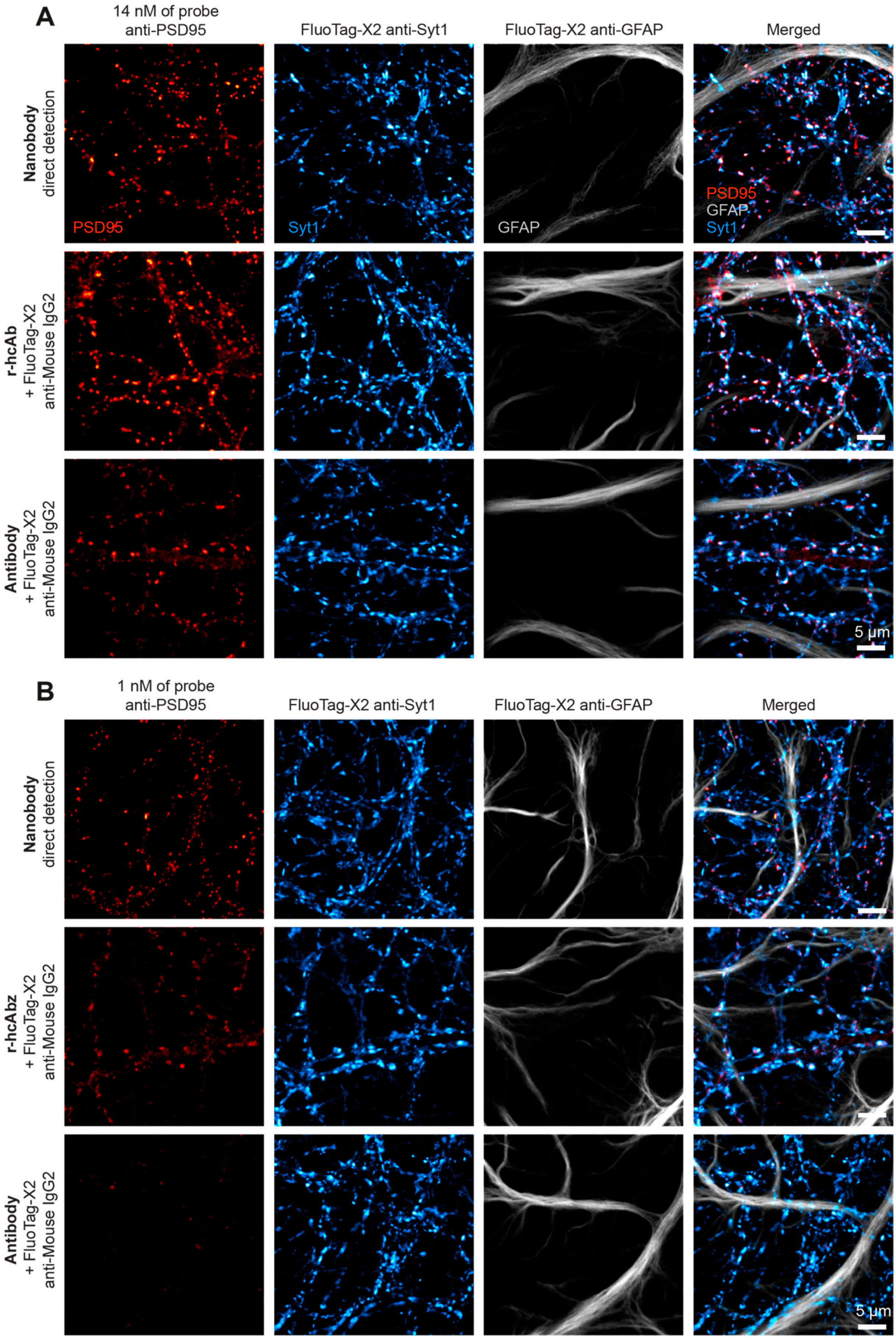

Figure S2: Comparison of NbPSD95 with an anti-PSD95 r-hcAb and a conventional antibody – related to Fig. 3. Figure shows separate channels used for creating overlays displayed in Fig. 3.

Table S1: Antibodies

Figure	Panel(s)	Product	Product number	Provider	Description	Application	Dilution/ Conc.
Fig. 2A		Monoclonal anti-FLAG HRP	A8592	Merck	HRP-conjugated anti-FLAG antibody (clone M2)	ELISA	1:5000
Fig. 2B	upper panels	FluoTag-X2 anti-PSD95 AZDye568	N3702-AF568-L	NanoTag Biotechnologies	Fluorophore-conjugated NbPSD95	IF, direct	1:500
Fig. 2C	upper left panel	Recombinant anti-PSD95 Antibody (R-hcAb), Mouse IgG1 Fc fusion	N3782	NanoTag Biotechnologies	NbPSD95 fused to mouse IgG1 Fc domain	WB, 1st Ab	1:1000
Fig. 2C	lower panels	Mouse anti-beta-Actin	251 011	Synaptic Systems	Monoclonal antibody	WB, 1st Ab	1:1000
Fig. 2C	upper right panel + lower panels	sdAb anti-MsIgG1 HRP	N2005-HRP	NanoTag Biotechnologies	HRP-conjugated anti-Mouse IgG1 nanobody	WB, 2nd Ab	1:4000
Fig. 2C	upper right panel	Recombinant anti-PSD95 Antibody (R-hcAb), Rabbit Fc fusion	N3783	NanoTag Biotechnologies	NbPSD95 fused to rabbit IgG Fc domain	WB, 1st Ab	1:1000
Fig. 2C	upper right panel	sdAb anti-RbIgG HRP	N2405-HRP	NanoTag Biotechnologies	HRP-conjugated anti-Rabbit IgG nanobody	WB, 2nd Ab	1:4000
Fig. 2D		FluoTag-X2 anti-PSD95 AZDye568	N3702-AF568-L	NanoTag Biotechnologies	Fluorophore-conjugated NbPSD95	IF, direct	1:500
Fig. 2D		FluoTag-X2 anti-GFAP Atto488	N3802-At488-L	NanoTag Biotechnologies	Fluorophore-conjugated anti-GFAP nanobody	IF, direct	1:500
Fig. 3A,<br>Fig. 3B	upper row	FluoTag-X2 anti-PSD95 AbberiorStar635P	N3702-Ab635P-L	NanoTag Biotechnologies	Fluorophore-conjugated NbPSD95	IF, direct<br>IF, direct	14 nM<br>1 nM
Fig. 3A,<br>Fig. 3B	middle row	Recombinant anti-PSD95 Antibody (R-hcAb), Mouse IgG2 Fc fusion	N3785	NanoTag Biotechnologies	NbPSD95 fused to mouse IgG2 Fc domain	IF, 1st Ab<br>IF, 1st Ab	14 nM<br>1 nM
Fig. 3A,<br>Fig. 3B	lower row	Monoclonal anti-PSD95 antibody	MABN68	Millipore	Monoclonal antibody (Mouse IgG2)	IF, 1st Ab<br>IF, 1st Ab	14 nM<br>1 nM
Fig. 3A, B	two lower rows	FluoTag-X2 anti-Mouse IgG2 AbberiorStar635P	N2702-Ab635P	NanoTag Biotechnologies	Fluorophore-conjugated anti-Mouse IgG2 nanobody	IF. 2nd Ab	1:500
Fig. 3		FluoTag-X2 anti-Syt1 AZDye568	N2302-AF568-L	NanoTag Biotechnologies	Fluorophore-conjugated anti-Synaptotagmin1 nanobody	IF, direct	1:500
Fig. 3		FluoTag-X2 anti-GFAP Atto488	N3802-At488-L	NanoTag Biotechnologies	Fluorophore-conjugated anti-GFAP nanobody	IF. direct	1:500

Table S1: Antibodies (continued)

Figure	Panel(s)	Product	Product number	Provider	Description	Application	Dilution/ Conc.
Fig. 4		FluoTag-X2 anti-PSD95 AbberiorStar635P	N3702-Ab635P-L	NanoTag Biotechnologies	Fluorophore-conjugated NbPSD95	IF, direct	1:500
Fig. 4		FluoTag-X2 anti-Syt1 AZDye568	N2302-AF568-L	NanoTag Biotechnologies	Fluorophore-conjugated anti-Synaptotagmin1 nanobody	IF, direct	1:500
Fig. 4		FluoTag-X2 anti-GFAP Atto488	N3802-At488-L	NanoTag Biotechnologies	Fluorophore-conjugated anti-GFAP nanobody	IF, direct	1:500
Fig. 5		FluoTag-X2 anti-PSD95 Sulfo-Cy3	N3702-SC3-L	NanoTag Biotechnologies	Fluorophore-conjugated NbPSD95	IHC, direct	1:500
Fig. 6		FluoTag-X2 anti-PSD95 AbberiorStar635P	N3702-Ab635P-L	NanoTag Biotechnologies	Fluorophore-conjugated NbPSD95	IHC, direct	1:500
Fig. 6		Recombinant anti-Synaptotagmin antibody	105 008	Synaptic Systems	Rabbit monoclonal recombinant antibody	IHC, 1st Ab	2 µg/mL
Fig. 6		FluoTag-X2 anti-Rabbit IgG AbberiorStar580	N2402-Ab580-L	NanoTag Biotechnologies	Fluorophore-conjugated anti-Rabbit IgG2 nanobody	IHC, 2nd Ab	1:500
Fig. 7		Recombinant anti-PSD95 Antibody (R-hcAb), Rabbit Fc fusion	N3783	NanoTag Biotechnologies	NbPSD95 fused to rabbit IgG Fc domain	IHC-P, 1st Ab	1 µg/mL
Fig. 7		Secondary anti-Rabbit Biotin	111-065-144	Jackson Immuno Research	Biotin-SP AffiniPure Goat Anti-Rabbit IgG	IHC-P, 2nd Ab	5 µg/ml
Fig. 7		Avidin-Biotin Complex (ABC)-HRP Kit	PK-4000	Vector Laboratories	ABC-HRP Kit	IHC-P, amplification	prepared according to manufacturer's instructions

Table S1: Antibodies (continued)

Figure	Panel(s)	Product	Product number	Provider	Description	Application	Dilution/ Conc.
Fig. S1A		Recombinant anti-PSD95 Antibody (R-hcAb), Mouse IgG1 Fc fusion	N3782	NanoTag Biotechnologies	NbPSD95 fused to mouse IgG1 Fc domain	WB, 1st Ab	1:1000
Fig. S1A		sdAb anti-MsIgG1 HRP	N2005-HRP	NanoTag Biotechnologies	HRP-coupled anti-Mouse IgG1 nanobody	WB, 2nd Ab	1:4000
Fig. S1B		Recombinant anti-PSD95 Antibody (R-hcAb), Mouse IgG2 Fc fusion	N3785	NanoTag Biotechnologies	NbPSD95 fused to mouse IgG2 Fc domain	WB, 1st Ab	1:1000
Fig. S1B		sdAb anti-MsIgG2 HRP	N2705-HRP	NanoTag Biotechnologies	HRP-coupled anti-Mouse IgG2 nanobody	WB, 2nd Ab	1:4000
Fig. S1C		Recombinant anti-PSD95 Antibody (R-hcAb), Rabbit Fc fusion	N3783	NanoTag Biotechnologies	NbPSD95 fused to rabbit IgG Fc domain	WB, 1st Ab	1:1000
Fig. S1C		sdAb anti-RbIgG HRP	N2405-HRP	NanoTag Biotechnologies	HRP-coupled anti-Rabbit IgG nanobody	WB, 2nd Ab	1:4000
Fig. S2A, Fig. S2B	1st rows	FluoTag-X2 anti-PSD95 AbberiorStar635P	N3702-Ab635P-L	NanoTag Biotechnologies	Fluorophore-conjugated NbPSD95	IF, direct<br>IF, direct	14 nM<br>1 nM
Fig. S2A, Fig. S2B	2nd rows	Recombinant anti-PSD95 Antibody (R-hcAb), Mouse IgG2 Fc fusion	N3785	NanoTag Biotechnologies	NbPSD95 fused to mouse IgG2 Fc domain	IF, 1st Ab<br>IF, 1st Ab	14 nM<br>1 nM
Fig. S2A, Fig. S2B	3rd rows	Monoclonal anti-PSD95 antibody	MABN68	Millipore	Monoclonal antibody (Mouse IgG2)	IF, 1st Ab<br>IF, 1st Ab	14 nM<br>1 nM
Fig. S2A, B	2nd/3rd rows	FluoTag-X2 anti-Mouse IgG2 AbberiorStar635P	N2702-Ab635P	NanoTag Biotechnologies	Fluorophore-conjugated anti-Mouse IgG2 nanobody	IF. 2nd Ab	1:500
Fig. S2		FluoTag-X2 anti-Syt1 AZDye568	N2302-AF568-L	NanoTag Biotechnologies	Fluorophore-conjugated anti-Synaptotagmin1 nanobody	IF, direct	1:500
Fig. S2		FluoTag-X2 anti-GFAP Atto488	N3802-At488-L	NanoTag Biotechnologies	Fluorophore-conjugated anti-GFAP nanobody	IF, direct	1:500

IF: Immunofluorescence; **IHC**: Immunohistochemistry; **IHC-P**: Immunohistochemistry on paraffin-embedded sections, **WB**: Western blot; **1st Ab:** Primary antibody; **2nd Ab**: Secondary antibody
